# Evaluation of Correlation between Temperature of IoT Microcontroller Devices and Blockchain Energy Consumption in Wireless Sensor Networks

**DOI:** 10.3390/s23146265

**Published:** 2023-07-10

**Authors:** Kithmini Godewatte Arachchige, Philip Branch, Jason But

**Affiliations:** Department of Telecommunications, Electrical, Robotics and Biomedical Engineering, Swinburne University, Melbourne 3122, Australia; pbranch@swin.edu.au (P.B.);

**Keywords:** blockchain, Internet of Things, energy, temperature, security, microcontrollers, low-powered, wireless, sensors

## Abstract

Blockchain technology is an information security solution that operates on a distributed ledger system. Blockchain technology has considerable potential for securing Internet of Things (IoT) low-powered devices. However, the integration of IoT and blockchain technologies raises a number of research issues. One of the most important is the energy consumption of different blockchain algorithms. Because IoT devices are typically low-powered battery-powered devices, the energy consumption of any blockchain node must be kept low. IoT end nodes are typically low-powered devices expected to survive for extended periods without battery replacement. Energy consumption of blockchain algorithms is an important consideration in any application that combines both technologies, as some blockchain algorithms are infeasible because they consume large amounts of energy, causing the IoT device to reach high temperatures and potentially damaging the hardware; they are also a possible fire hazard. In this paper, we examine the temperatures reached in devices used to process blockchain algorithms, and the energy consumption of three commonly used blockchain algorithms running on low-powered microcontrollers communicating in a wireless sensor network. We found temperatures of IoT devices and energy consumption were highly correlated with the temperatures reached. The results indicate that device temperatures reached 80 °C. This work will contribute to developing energy-efficient blockchain-based IoT sensor networks.

## 1. Introduction

Blockchain technology and the Internet of Things (IoT) are innovative technologies that are proving useful in industries as diverse as healthcare, automotives, finance, and supply chain logistics [1]. Security features including the decentralised nature of blockchain technology may address many cybersecurity issues in those respective industries. Aged care is one area that has recently started using IoT technology [2]. Aged care is primarily concerned with the health and wellbeing of elderly people. Lack of information transparency and data leakage in aged care could be life-threatening. The IoT industry uses low-powered microcontroller devices to develop ambient assisted living systems for the aged care industry, and potential data corruption or miscalculation can put lives at risk [3].

The combination of blockchain and IoT technologies creates intrinsic benefits. Securing IoT end devices, which has been a challenge, is one of the key benefits. Blockchain technology has also benefited from microcontroller developments in the emergence of energy-efficient IoT devices and blockchain algorithms [4]. As low-powered microcontroller devices are portable and cost-effective, the IoT industry uses these microcontroller devices to develop and manufacture sensor-based IoT devices. Connected IoT end devices may process a large amount of sensitive sensor data, and it may be difficult to locate the source of a data leak in an event of a cyber threat [4]. 

Blockchain technology may help ease the potential security and scalability issues of sensor networks. Blockchain technology adds another layer of security to data transmission, and makes it more difficult for cyber attackers to gain access. Additionally, it brings transparency to network access [5]. Alongside increasing the trust between parties involved, the blockchain can have other benefits, including the elimination of specialist gateways solely intended to secure IoT sensor networks [6].

However, the integration of blockchain technology and microcontroller technologies can be a challenge due to energy consumption requirements [7]. Blockchains require significant computational resources, and thus consume additional energy in energy-constrained sensor devices, leading to higher temperatures. These higher temperatures may damage hardware devices and cause possible fire hazards. Fire hazards can put lives at risk. Understanding potential device temperature levels will contribute to preventing possible fire hazards. Additionally, the size and complexity of the blockchain network can also play a role in the energy consumption and temperature variations of hardware devices. A larger network with more nodes and transactions requires more energy to maintain and process [7].

In this paper, we analyse the correlation between the blockchain energy consumption and temperature of low-powered IoT devices using Raspberry Pi devices and three commonly used blockchain algorithms. Additionally, we discuss how the temperature of IoT devices that process blockchain algorithms may possibly influence the energy consumption of low-powered microcontroller devices [8]. This paper will contribute towards developing more energy-efficient blockchain algorithms and IoT hardware devices. The paper is structured as follows. In Section 2, we discuss blockchain technology and its energy consumption. In Section 3, we look at related work. Section 4 outlines our methodology, while Section 5 presents our results and evaluation. Section 6 concludes the paper and outlines future work we plan to carry out.

## 2. Blockchain Technology and Energy Consumption

Blockchain technology gained public recognition with the first blockchain algorithm, Bitcoin, which was established in 2008. In the last decade, blockchain technology has developed considerably, with a wide range of applications including Ethereum, Monero, Hydrachain, Duino Coin and Hyperledger Fabric [8]. Blockchain technology was invented as an information security solution for cryptocurrency, operating as a digital ledger system. However, researchers have realised that blockchain technology holds the potential to address many cybersecurity issues beyond cryptocurrency [9]. 

A blockchain forms a shared network among end devices which are called as blockchain nodes. There are three main different blockchain networks. 

### 2.1. Public Blockchain Networks

**Figure 1:**
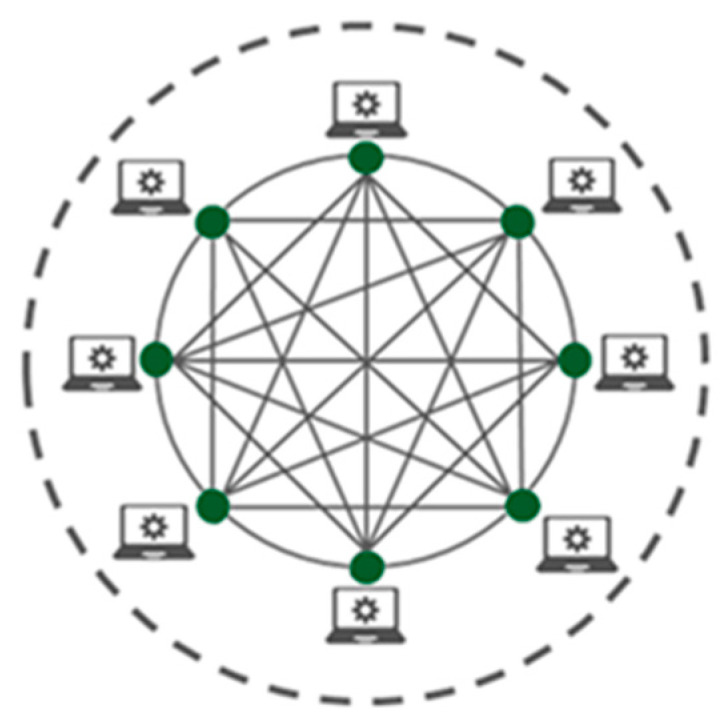
Public blockchain network.

Public blockchain networks provide unrestricted user access for all blockchain users to the blockchain network and security features. These public blockchain networks are called permissionless blockchain networks [9]. Users can read, write or alter transactions as per their requirements. These types of blockchain networks are self-governed networks that allow users to use security features such as encryption, time stamps, anonymity, and hashes. Figure 1 shows the architecture of a public blockchain network [9]. The green-coloured dots indicate the users who have access to the blockchain network and services. As per Figure 1, all users have access to the blockchain network and its services in public blockchain networks.

### 2.2. Private Blockchain Networks

**Figure 2:**
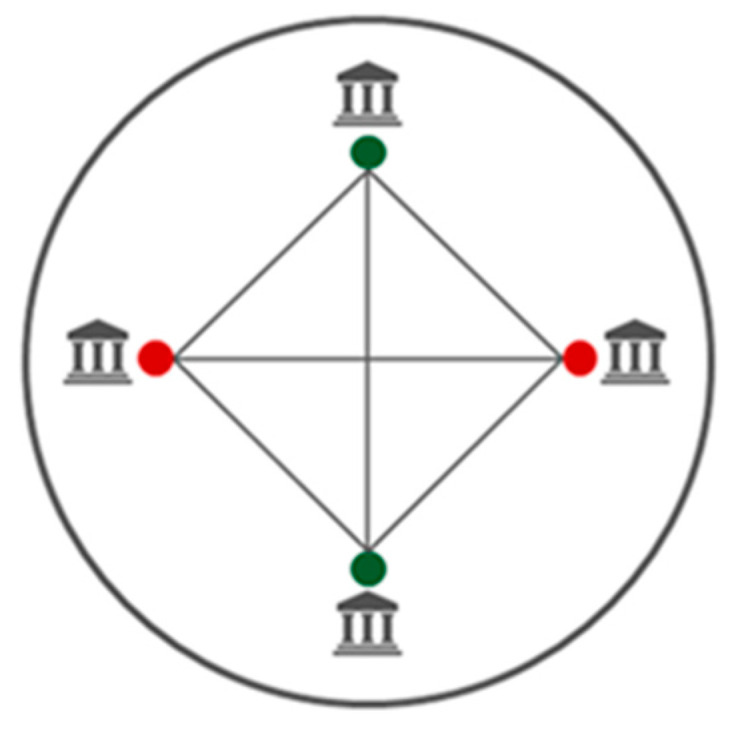
Private blockchain network.

Private blockchain networks provide restricted access wherein only authorised users can have access. Participants can only join these private blockchain networks through an invitation, and are required to verify their identification [10]. User validations are controlled by automated smart contracts [9]. Private blockchain networks are called permissioned blockchain networks. Additionally, only selected or authenticated users can access the shared ledger. Figure 2 shows the architecture of a private blockchain network [9]. The green-coloured dots in Figure 2 indicate the users who have access to the blockchain network and services, and the red-coloured dots indicate the users who do not have access to the blockchain network and services. As Figure 2 shows, only authorised users have access to the blockchain network and its services in private blockchain networks.

### 2.3. Hybrid Blockchain Networks

**Figure 3:**
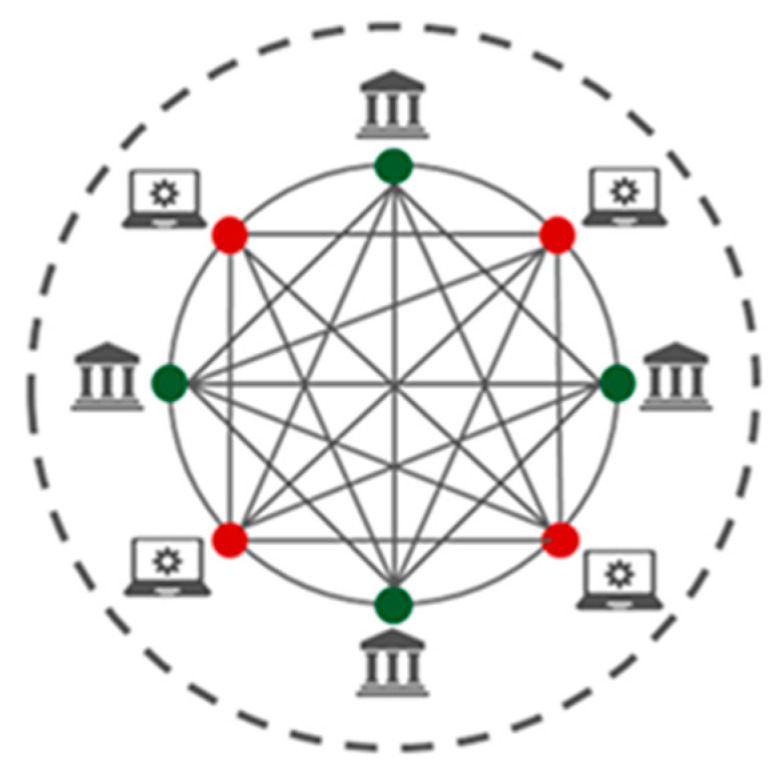
Hybrid blockchain network.

Hybrid blockchain networks are a combination of private blockchain networks and public blockchain networks. They blend essential blockchain components and protocols of both private and public blockchain networks. Any blockchain user can access the blockchain network, but only certain users can access all security features and services [11]. Hybrid blockchains are owned by a private user who can grant access to the public via smart contracts. The structures of hybrid blockchain networks are highly customisable, and users can choose their desired type of transactions. Figure 3 shows the architecture of a hybrid blockchain network [12]. As Figure 3 indicates, the green-coloured dots indicate users who have access to the blockchain network and services. The red-coloured dots show the users who do not have access to the blockchain network and its services. Figure 3 shows that every user has access to the blockchain network, but only certain users have access to blockchain network services. 

Blockchain technology has primarily been designed for computers with high processing power. However, with the development of the IoT industry, some researchers have begun to focus on energy-efficient blockchain solutions for low-powered IoT devices [13]. In particular, the aged care sector has started using IoT sensor-based devices to develop ambient assisted living systems [14]. As the aged care sector uses low-powered microcontroller devices to implement ambient assisted living systems and health sensor networks, blockchain algorithms must be energy-efficient [14]. The development of blockchain algorithms for IoT low-powered devices is an increasingly active research area [15]. 

**Figure 4:**
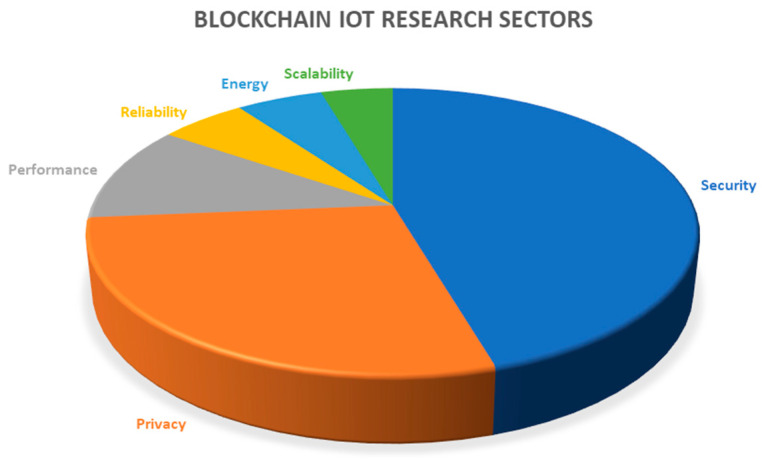
Popular blockchain IoT research sectors [7].

However, very little research has been conducted to identify blockchain energy consumption variations in low-powered microcontroller devices. Blockchain energy consumption in microcontroller devices is a significant factor that needs to be evaluated, as IoT end nodes are expected to run for long periods without battery replacement. We will use this work to help develop energy-efficient blockchain sensor networks [16]. Figure 4 shows which blockchain research areas are most popular in the IoT industry. As Figure 4 shows, most of the research that has been conducted targets blockchain security and privacy. There has been very little research conducted to evaluate blockchain energy consumption.

Modern, IoT solutions also focus on renewable energy solutions. As a result of this, low-power IoT devices and energy solutions have been significantly improved by researchers [17]. Understanding the energy requirements of blockchain technologies will contribute to the use of suitable renewable energy sources for low-powered sensor networks. Different blockchain algorithms may consume different amounts of energy, and energy consumption is an important consideration when choosing which blockchain algorithm to use on low-powered microcontroller devices. Low-powered IoT device performance and energy consumption may also be correlated. If the energy consumption of a particular blockchain algorithm is high, the performance of the microcontroller device may be negatively affected [18]. 

To establish maximum blockchain functionality in a microcontroller device, the utilisation of energy may be necessary. Understanding the energy consumption of low-powered microcontrollers and different blockchain algorithms is important, because it affects battery life and microcontroller performance. This work contributes to our collective understanding of the energy consumption of battery-operated IoT devices. In this paper, we evaluate the correlation between blockchain temperature variations and blockchain energy consumption in IoT low-powered microcontroller devices. We discuss related works in the next section.

## 3. Related Work

According to She, W. et al., IoT technologies have attracted exponentially growing interest since cyber-physical systems started using the Internet of Things [16]. Since IoT devices are usually connected to the internet, cybersecurity issues can emerge. Blockchain technology is one of the security solutions that provides security features including data encryption and secure storage facilities. Additionally, blockchain technology provides digital signatures, timestamps, and hash functions [16]. This paper focuses on how to strengthen the privacy and security of IoT devices using blockchain technology. The authors have reviewed advanced security requirements for IoT-based cyber-physical systems [16]. Resource-constrained IoT devices using low-powered sensors for data communication and blockchain technology can be used to address user privacy and information integrity concerns [16]. According to She, W. et al., though blockchain technology is a reliable security solution, the utilisation of computational resources and power is still a contentious matter [16].

According to Johannes Sedlmeir et al., the power consumption of blockchain algorithms is a key area that needs to be addressed [17]. For the sustainable deployment of blockchain networks in businesses, the power consumption of blockchain networks is crucial. This paper summarises the power consumption of Bitcoin blockchain networks. In this paper, the authors provide a comprehensive overview of Bitcoin blockchain networks’ periodic power consumption [17]. The authors have evaluated both recently developed Bitcoin blockchain algorithms and older blockchain algorithms that are not so common now. The main focus of the paper is to address the requirements of blockchain applications beyond cryptocurrency. As the authors have emphasised, numerous blockchain algorithms have been modified significantly, but very little research has been conducted to evaluate the power consumption of blockchain networks [17]. The power consumption of blockchain algorithms can vary based on device performance and the number of transactions [17]. As per the evaluation, proof of work (PoW) blockchain algorithms use less power compared to proof of stake (PoS) blockchain algorithms [17].

According to Abigael Okikijesu Bada et al., the use of blockchain technology and its services in industries such as the Internet of Things, supply chain, and healthcare, has significantly increased [18]. Additionally, the energy consumption of blockchain algorithms is concerned with the use of blockchain services [18]. In particular, the need for green and sustainable energy sources to power blockchain networks attracted attention. As the authors have emphasised, blockchain technology tends to have high energy consumption depending on the consensus mechanism [18]. This paper provides a comprehensive review of the energy consumption of various consensus mechanisms, and contributes to developing more sustainable blockchain-enabled systems [18]. As the authors have mentioned, reducing the impact of the high energy consumption of blockchain algorithms may alleviate unnecessary wastage of resources. Although modern technological developments follow the principles of green IT and use energy-efficient resources, blockchain technology still consumes large amounts of energy. This paper reviews 18 consensus mechanisms and highlights energy-efficient blockchain algorithms [18]. 

According to Marko Hölbl et al., blockchain technology provides distributed and decentralised network features for sensor networks [19]. Therefore, the need for a central authority to authenticate user access and sensor data transmission may be optional [19]. All data transactions are secured using encryption algorithms. According to this paper, healthcare has started using blockchain applications to secure health data [19]. Health sensor networks use blockchain applications to maintain the authenticity and integrity of electronic health records [19]. The main focus of the research is to find potentially reliable and sustainable blockchain applications to face different cyber challenges in the healthcare sensor network environment. The authors claim that blockchain technology has opened up new research paths, including the evaluation of blockchain power consumption and hardware resource utilisation [19]. The authors highlight that some modern blockchain applications are integrated with biometric authentication to authenticate users and avoid possible unauthorised user access, as well as using secure block architecture to transmit encrypted data. Because of the additional security features, the power consumption of blockchain algorithms may impact hardware performance utilisation. Therefore, it is necessary to understand blockchain-related security capabilities and performance capabilities. We discuss the sensor network system design and its architecture in the next section.

## 4. Methodology

The methodology of this research is a blended methodology with experimental results and quantitative analysis. A test bed was used to collect experimental power consumption and device temperature data. We have used statistical software tools to evaluate the power consumption and temperature data that we have collected from our test bed [20]. To evaluate performance and measure the energy usage and device temperature of networked devices running different blockchain algorithms, we deployed a physical IoT sensor network using low-powered devices. The same testbed was used to deploy a number of blockchain algorithms [21]. In this section, we describe our testbed.

### 4.1. Sensor Network Architecture

The testbed system design and architecture are based on a blockchain-based wireless sensor network that has been implemented using Raspberry Pi model 3B devices [22]. The research methodology is based on an experimental blockchain sensor network prototype and generates actual results in a lab environment. The purpose of this network prototype is to measure blockchain energy consumption in low-powered microcontroller devices [23]. The main contribution of this paper is to identify energy-efficient blockchain algorithms and use them to implement blockchain-based health sensor networks in aged care facilities. 

**Figure 5:**
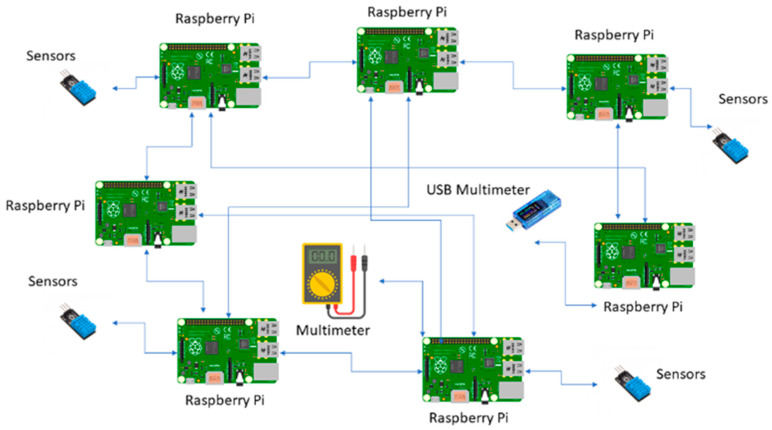
Sensor network architecture.

Seven Raspberry Pi devices have been used as blockchain end nodes to implement the network prototype. Additionally, we have installed Hydrachain, Monero, and Duino coin blockchain applications on the Raspbian Linux 32-bit version to collect energy consumption and temperature data. Figure 5 shows the architecture of the blockchain-based sensor network prototype [24].

Each Raspberry Pi device acts as a blockchain node, and data have been transmitted over the network using wireless TCP /IP protocols. We connected a digital multimeter and a USB digital multimeter to measure the energy consumption of microcontrollers [25]. Additionally, to collect temperature data, we have created a python-based code to run on each Raspberry Pi device while running blockchain applications, and have integrated a python-based code with the Linux vcgencmd tool to fetch device temperature data [26]. The energy consumption and temperature levels of each blockchain node have been analysed individually. In the next section, we discuss the results that we have acquired from the blockchain network prototype experiments.

### 4.2. Resources

In this section, we describe the resources that we have used to implement our testbed. We used software and hardware resources to develop the blockchain-based sensor network prototype.

#### 4.2.1. Raspberry Pi

**Figure 6:**
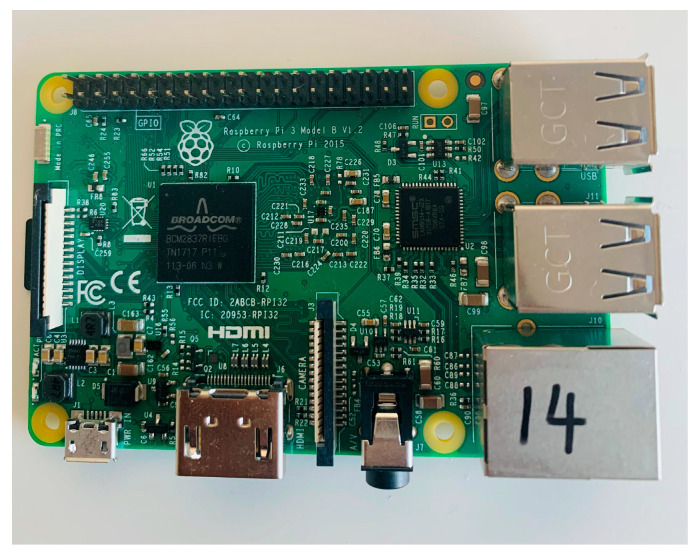
Raspberry Pi 3B device.

Raspberry Pi device series are ARM-based devices that are powered by ARM Linux operating systems such as Raspbian, Ubuntu, Manjaro, and RetroPi. These devices can be used for networking purposes and prototype developments [27]. Network connections can be established using a web panel, and this web panel allows users to manage the bridge library. These microcontroller devices are open-source devices. Figure 6 shows the Raspberry Pi 3B device that we used to develop the sensor network prototype [28]. 

The Raspberry Pi device series are single board computers and encompass a range of different models starting from model 04 to model zero. All these Raspberry Pi models are capable of running a Linux-based Raspbian operating system. Different models contain different RAM and processing capacity such as 512 MB and 1 GB. Raspberry Pi devices consist of 40 pin headers for connecting sensor devices, and a wireless LAN for networking purposes. Raspberry Pi devices are powered by ARM cortex CPUs [22]. 

#### 4.2.2. Hydrachain Blockchain Algorithm

The Hydrachain blockchain platform was designed as an open-source blockchain solution for industrial organisations. Early Hydrachain solutions were used as private blockchain platforms by organisations [23]. Modern Hydrachain blockchain solutions have been designed to achieve the requirements of both private and public blockchains. Hydrachain platforms support all widely used operating systems, including Linux and Microsoft Windows [23]. The Hydrachain platform uses the public address as the principal node connection protocol, and keeps a copy of all block transaction lists. Blockchain user metadata are stored securely. Users can access several Hydrachain networks with the same user account. Developers can use system configurations, per the requirement. Hydrachain provides a default configuration file, and the blockchain platform allows developers to change the configurations file if developers wish to proceed with their own configurations. The Hydrachain platform uses the hash function to secure blocks, and uses node verification protocols to identify legitimate block nodes [23]. 

#### 4.2.3. Monero Blockchain Algorithm

The Monero blockchain algorithm is a decentralised blockchain application that uses a publicly distributed ledger system. Monero blockchain technology provides anonymity and fungibility for data transactions over the blockchain network. Additionally, Monero blockchain promises that third parties cannot decipher Monero blockchain transactions and have no access to transaction histories [24]. The Monero blockchain algorithm uses ring signatures for enhancing blockchain network security. Ring signatures allow message signing using a set of public keys instead of one single public key. Ring signatures are a lightweight anonymous authentication protocol. The verifier is able to verify these ring signatures using one of their public keys. These public keys are called ring members [24]. 

#### 4.2.4. Duino Coin Blockchain Algorithm

The Duino coin blockchain algorithm is known as DUCO-S1. It is an open-source blockchain algorithm. This algorithm has been specially designed to target low-powered microcontroller devices and single-board computers such as Raspberry Pi, Arduino and ESP devices [10]. Duino coin application uses the “Kolka System” to maintain the under-power transactions without causing difficulty [10]. This blockchain algorithm uses SHA-1 encryption to provide data security. Duino coin contains a decentralised ledger system and provides privacy to blockchain users [10]. We provide an overall evaluation of the results in the next section. 

## 5. Results and Evaluation

We compared the energy consumption data and temperature data of Hydrachain, Monero and Duino coin blockchain algorithms in a wireless sensor network environment. With the high temperature levels of blockchain algorithms, high energy consumption levels can be expected. The lower and upper bounds of these blockchain energy consumption levels need to be analysed to identify which blockchain algorithms are more energy-efficient [29]. In the next section, we discuss temperature data evaluation. 

### 5.1. Temperature Data Evaluation

Blockchain temperature is an important factor in monitoring the blockchain energy consumption behaviours of low-powered microcontroller devices. Health sensors and sensor-based aided devices are battery-powered devices that can be damaged by high temperature [29]. Particularly, if health sensors are damaged or corrupted due to high temperatures, health data can be lost [30]. Additionally, in high-temperature conditions, health sensors may observe false data. Therefore, analysing the temperature levels of the hardware devices that we use to process blockchain algorithms is crucial for implementing blockchain-based health sensor networks [31].

**Figure 7:**
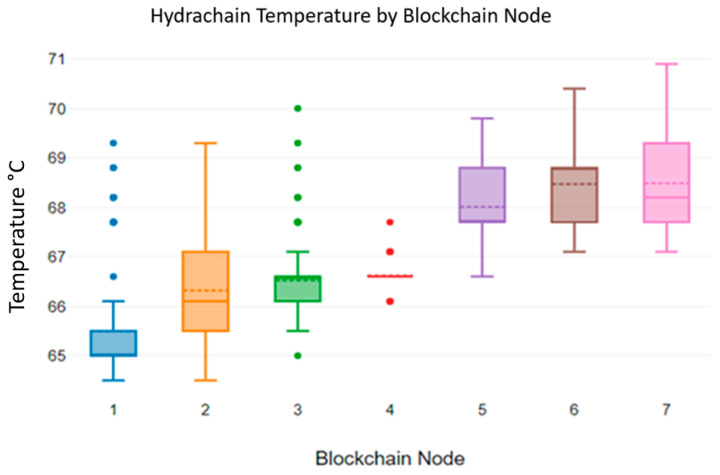
Hydrachain blockchain nodes’ temperature levels.

We have individually analysed how device temperature varies based on three blockchain algorithms. Figure 7 shows the temperature variations of Hydrachain blockchain nodes. 

Figure 7’s results indicate that the temperature of each individual blockchain node deviates from 65 °C to 71 °C. 

**Figure 8:**
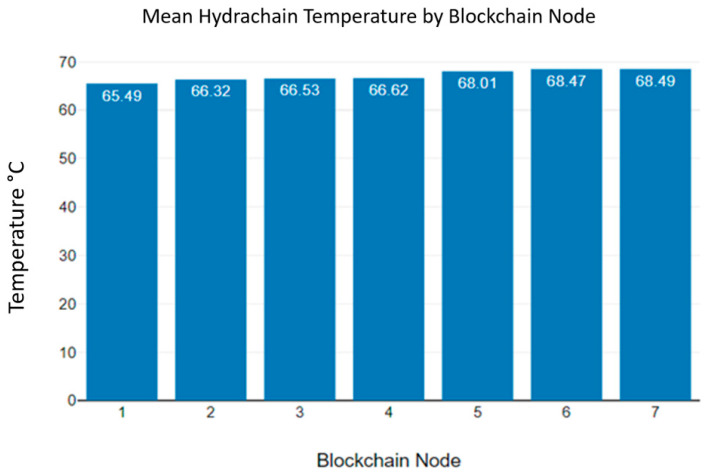
Hydrachain blockchain nodes’ mean temperature levels.

**Figure 9:**
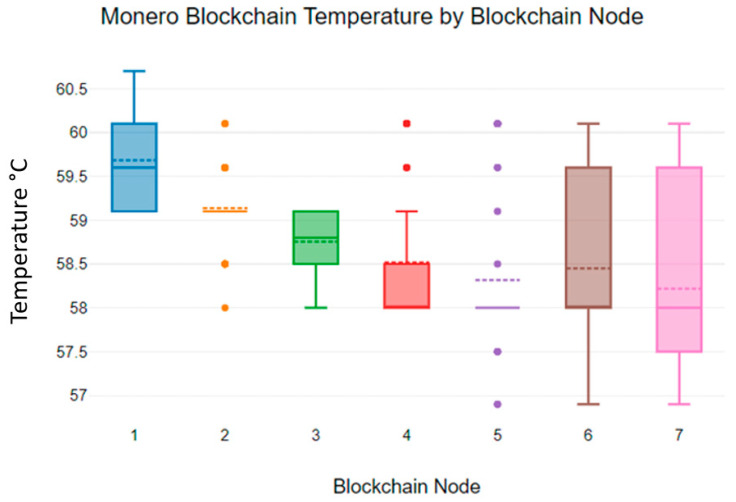
Monero blockchain nodes’ temperature levels.

Figure 8 shows the mean Hydrachain temperature of the blockchain nodes. The maximum mean temperature recorded at the first blockchain node was 65.49 °C. Additionally, the highest at the seventh blockchain node was recorded as 68.49 °C. High temperatures may damage the hardware components of microcontroller devices [32]. Figure 9 displays the Monero temperature variation observed while transmitting data blocks. 

Figure 9’s results show that the Monoro blockchain algorithm temperature values are lower than those of the Hydrachain blockchain algorithm. The temperature values of the Monero blockchain algorithm deviate from 57 °C to 60.5 °C. This may result in lower energy consumption in Monero blockchain sensor networks [33].

**Figure 10:**
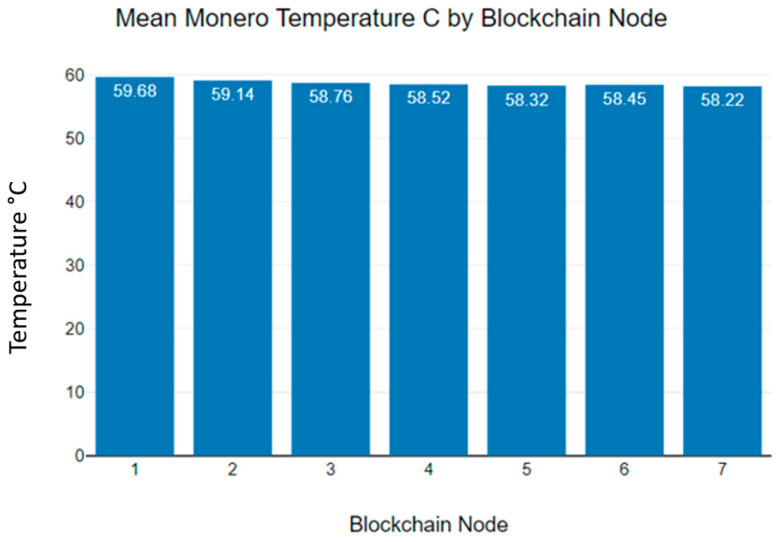
Monero blockchain nodes’ mean temperature levels.

Figure 10 shows the mean Monero temperature variations in individual blockchain nodes. As Figure 10 indicates, the maximum mean temperature recorded at the first blockchain node was 59.68 °C, and the minimum was recorded at blockchain node 7, at 58.22 °C.

**Figure 11:**
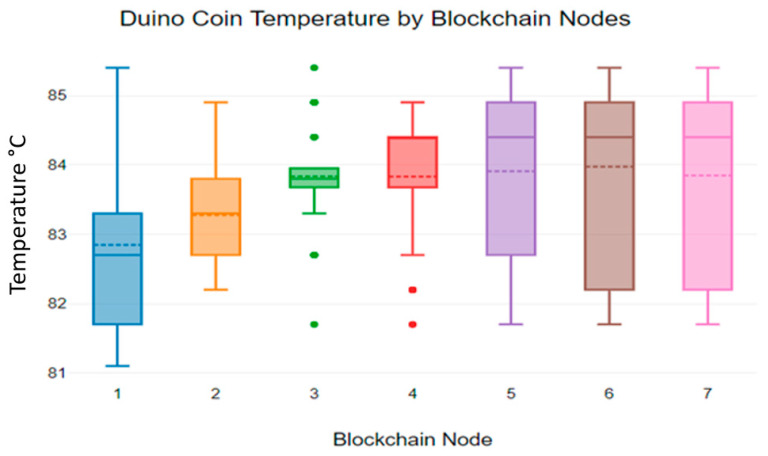
Duino coin blockchain nodes’ temperature levels.

Additionally, we analysed the temperature variations in the Duino coin blockchain algorithm, and Figure 11 shows the temperature values measured while using the Duino coin blockchain algorithm to transmit sensor data [34]. 

As Figure 11 shows, the Duino coin blockchain algorithm shows the highest temperature values while transmitting sensor data, compared to the Hydrachain and Monero blockchain algorithms. According to Figure 11, the Duino coin blockchain temperature values deviate from 81 °C to 85 °C. This indicates that the heat of the Duino coin blockchain algorithm is typically high when compared to other two blockchain applications [35]. 

**Figure 12:**
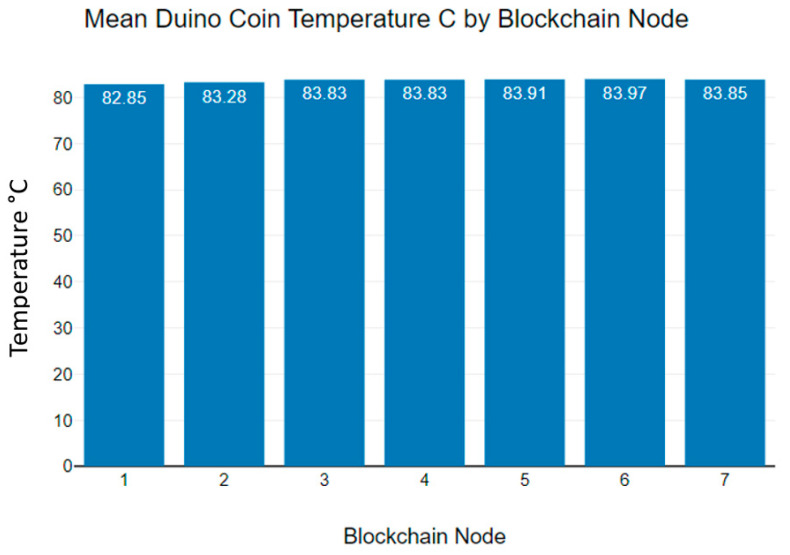
Duino coin blockchain nodes’ mean temperature levels.

Figure 12 shows the mean Duino coin temperature variations observed while transmitting data. As the graph indicates, the mean temperature of all seven blockchain nodes is over 80 °C. 

**Figure 13:**
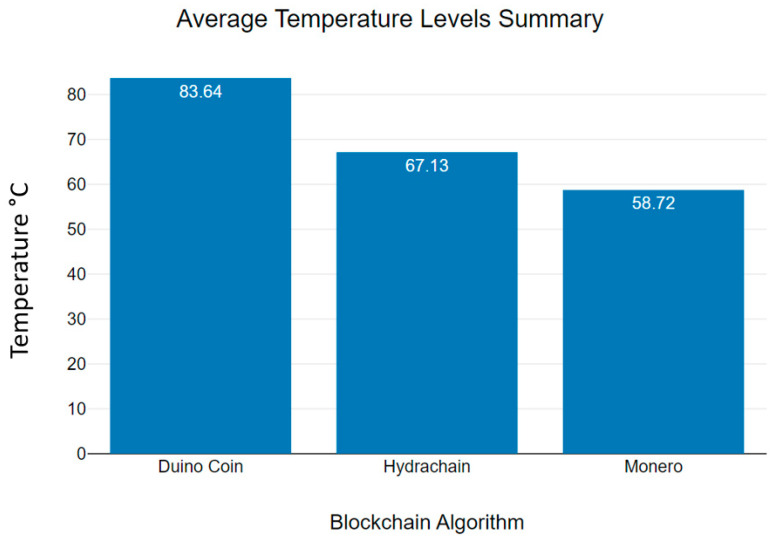
Average temperature level summary.

Figure 13 shows us the average temperature of microcontroller devices that were used to process three blockchain algorithms [36]. Additionally, Table 1 shows an overall summary of the mean temperature values that the hardware devices indicated while processing Hydrachain, Monero and Duino coin blockchain algorithms [37]. Based on this analysis, we can emphasise that the temperature levels of the Monero blockchain algorithm are lower than those of the Hydrachain and Duino Coin blockchain algorithms in a wireless sensor network environment. Additionally, as Table 1 indicates, device temperature levels can vary for different reasons, such as data transmission and receiving time periods, CPU core usage levels, device location, and environmental factors such as heat [38].

An analysis of the device temperature levels that are used to process each blockchain algorithm is significant for identifying the best method of thermal management for different blockchain architectures [39]. Additionally, this may help us to understand the energy consumption of different blockchain algorithms in low-power microcontroller devices. In particular, wireless health sensor networks use battery power to power up sensors and microcontroller devices [40]. Therefore, temperature levels are significant parameters for analysing blockchain energy consumption in low-powered IoT devices; we discuss energy consumption variations in the next chapter.

### 5.2. Energy Consumption Data Evaluation

**Figure 14:**
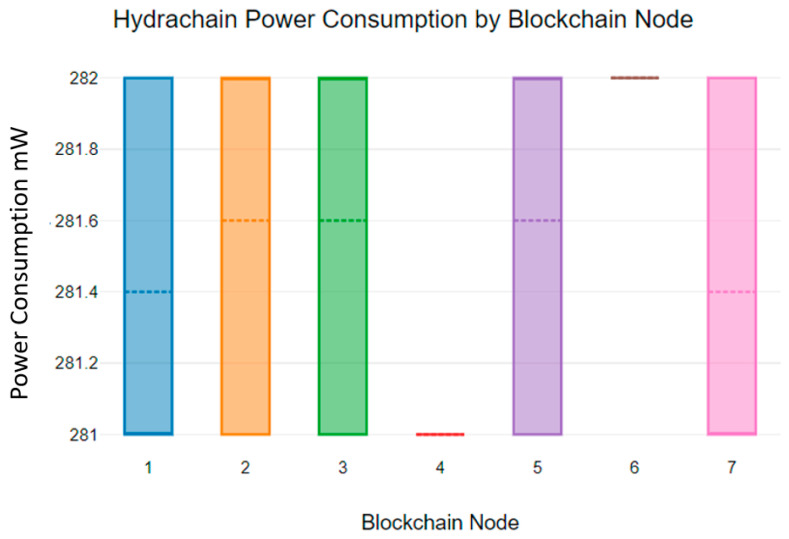
Hydrachain blockchain power consumption.

The comparison of blockchain algorithms’ power consumption is highly significant for health sensor networks. Most medical IoT devices and sensor networks are battery-powered devices and are expected to survive for extended periods without battery replacement [41]. Therefore, battery consumption is an important parameter for blockchain energy consumption analysis. Figure 14 shows the energy consumption of the Hydrachain blockchain algorithm in Raspberry Pi 3B devices.

**Figure 15:**
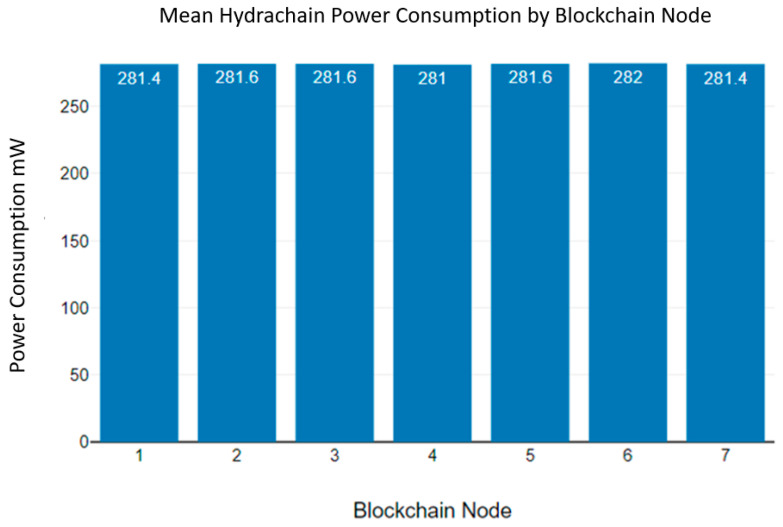
Hydrachain blockchain mean power consumption.

As Figure 14 indicates, blockchain nodes consumed 281 mW to 282 mW while running the Hydrachain blockchain algorithm. Additionally, as Figure 15 indicates, the mean Hydrachain blockchain power consumption deviates from 281 mW to 282 mW.

As an example, the fourth blockchain node of Hydrachain has a mean power consumption of 281 mW. We used 9 V batteries to power the microcontroller devices and 9 V batteries containing 550 mAh. This means a 9 V battery can power up the blockchain node for up to 1.95 h continuously.

**Figure 16:**
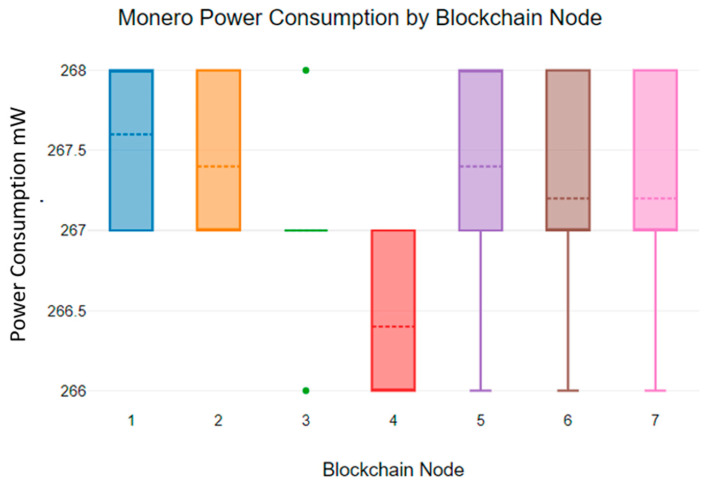
Monero blockchain power consumption.

Figure 16 shows the power consumption of the Monero blockchain algorithm. According to Figure 16, the power consumption of the Monero blockchain algorithm deviates from 266 mW to 268 mW. As Figure 16 indicates, the Monero blockchain algorithm consumes a small amount of energy compared to the Hydrachain blockchain algorithm.

**Figure 17:**
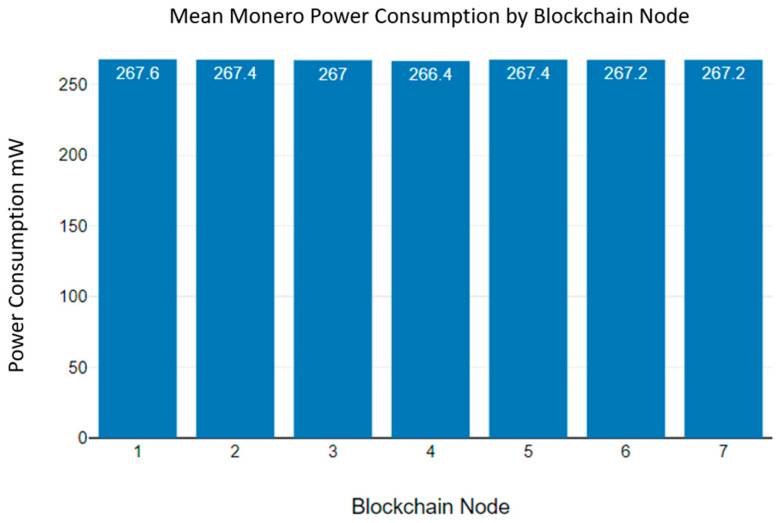
Monero blockchain mean power consumption.

Figure 17 shows the mean Monero power consumption of the blockchain nodes. According to Figure 17, a 9 V battery can power a blockchain node for 2 h. 

**Figure 18:**
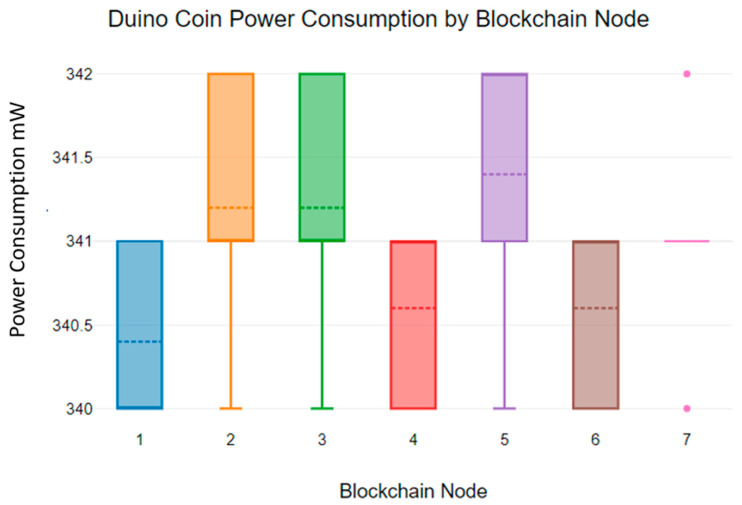
Duino coin blockchain power consumption.

**Figure 19:**
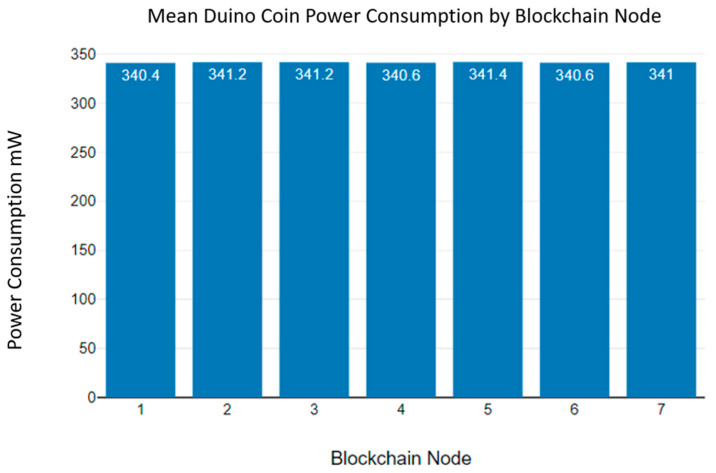
Duino coin blockchain mean power consumption.

Figure 18 and Figure 19 show the power consumption of the Duino coin blockchain algorithm.

According to Figure 18, the power consumption of the Duino coin blockchain algorithm deviates from 340 mW to 342 mW. Additionally, Figure 19 shows the mean power consumption of the Duino coin blockchain algorithm. According to Figure 19, the mean power consumption is 341 mW.

A 9 V battery only can provide power to a Duino coin blockchain node for up to 1.6 h. As the Duino Coin energy consumption graphs show, the power consumption of the Duino coin blockchain algorithm is significantly higher than that of the Monero and Hydrachain blockchain algorithms [42].

**Figure 20:**
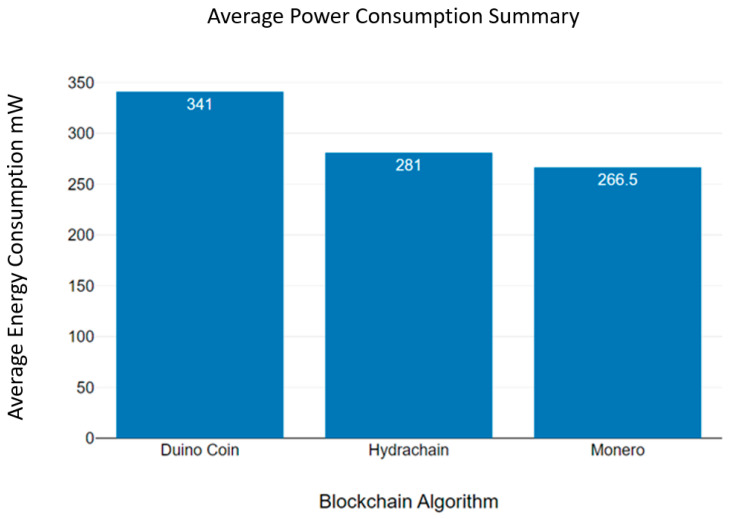
Average power consumption summary.

Figure 20 shows us the average power consumption summary of three blockchain algorithms. According to Figure 20, the Duino coin blockchain algorithm recorded the highest power consumption, and the Monero blockchain algorithm recorded the lowest. Additionally, Table 2 presents an overall summary of the mean power consumption of the Hydrachain, Monero and Duino coin blockchain algorithms [43]. 

Based on the analysed blockchain power consumption variations, we can emphasise that the Monero blockchain algorithm consumes the least energy, compared to Hydrachain and Duino coin algorithms, in a wireless sensor network environment. The results of Table 2 show the different power consumption levels of each node [44]. This indicates that the energy consumption of blockchain networks can be changed. The block transmission rate, block exchange periods, and hardware CPU core level performance are possible reasons for the different energy consumption levels [45]. Additionally, we can highlight that the average temperature levels and average energy consumption summaries are correlated. In the next chapter, we discuss our conclusions and possibilities for future research. 

## 6. Conclusions and Future Research

Blockchain technology and IoT-based low-powered microcontroller devices are emerging research areas. Blockchain technology has considerable potential in securing IoT low-powered sensor networks [46]. However, the integration of blockchain technology and IoT technologies is one of the key research areas that need to be considered. The integration of these technologies may enhance the wellbeing of elderly people [47] Blockchain technology represents an ideal opportunity for the aged care industry to prevent potential harmful threats and protect the elderly generation [47]. 

Currently, little research identifying the energy requirements of blockchain technology in low-powered IoT microcontroller devices has been published [48]. We have analysed the correlation between blockchain temperature and energy consumption data based on three blockchain algorithms to compare variations in energy requirements. Based on this research, we can highlight that there is a correlation between the energy consumption and temperature levels of blockchain nodes [48]. We noted that these algorithms consume large amounts of energy and generate potentially damaging and dangerously high temperatures [48]. This is an important consideration in the development of energy-efficient IoT blockchain-based low-powered sensor networks [49]

The integration of blockchain and IoT technologies may open new research avenues. Using renewable energy for low-powered IoT sensor networks may be a new research direction [49]. Another possible research area is the development of energy-efficient blockchain algorithms and microcontroller hardware devices [49]. In addition, blockchain networks’ performance on microcontroller devices and the scalability limitations of blockchain sensor networks are future research topics that we will address as part of our ongoing research. Finally, blockchain energy analysis may be significant in addressing current and future research issues in the IoT industry [49].

## Figures and Tables

**Table 1:** Blockchain algorithm mean temperature variations in blockchain nodes.

Blockchain Node	Hydrachain	Monero	Duino Coin
Node 01	65.49 °C	59.68 °C	82.85 °C
Node 02	66.32 °C	59.14 °C	83.28 °C
Node 03	66.53 °C	58.76 °C	83.83 °C
Node 04	66.62 °C	58.52 °C	83.83 °C
Node 05	68.01 °C	58.32 °C	83.91 °C
Node 06	68.47 °C	58.45 °C	83.97 °C
Node 07	68.49 °C	58.22 °C	83.85 °C

**Table 2:** Blockchain algorithm mean power consumption of the blockchain nodes.

Blockchain Node	Hydrachain	Monero	Duino Coin
Node 01	281.4 mW	267.6 mW	340.4 mW
Node 02	281.6 mW	267.4 mW	341.2 mW
Node 03	281.6 mW	267 mW	341.2 mW
Node 04	281 mW	266.4 mW	340.6 mW
Node 05	281.6 mW	267.6 mW	341.4 mW
Node 06	282 mW	267.2 mW	340.6 mW
Node 07	281.4 mW	267.2 mW	341 mW

## Data Availability

All datasets generated during the study are available upon request from the primary author.
